# Ultrasound-Assisted Extraction, Followed by Gas Chromatography–Mass Spectrometry for the Simultaneous Quantification of Ethinyl Estradiol and Drospirenone in Contraceptive Formulations

**DOI:** 10.3390/molecules28134978

**Published:** 2023-06-25

**Authors:** Javier Peña, Iria González-Mariño, José L. Pérez Pavón

**Affiliations:** Department of Analytical Chemistry, Nutrition and Bromatology, Faculty of Chemical Sciences, 37008 Salamanca, Spain; javierpena@usal.es (J.P.); jlpp@usal.es (J.L.P.P.)

**Keywords:** ethinyl estradiol, drospirenone, contraceptive tablets, gas chromatography, mass spectrometry

## Abstract

Contraceptive tablets typically contain a combination of two synthetic versions of an estrogen and a progestogen, which work together to inhibit the ovulation process. An accurate and precise quantification of these components is essential for contraceptive producers. In this study, we have developed the first gas chromatography–mass spectrometry (GC–MS) method for the simultaneous quantification of 17α-ethinyl estradiol (EE) and drospirenone (DP) in contraceptive formulations. Under the final working conditions, analytes were extracted from the solid by ultrasound-assisted extraction (15 min) in methanol. The resulting suspension was diluted in ethyl acetate, subjected to centrifugation and, finally, the supernatant was directly injected into the GC–MS system. No derivatization reagents were utilized. To correct for instrumental variations, calibration was performed using the internal standard method, with cholesterol as the internal standard. A good linearity was achieved throughout the calibration range for both EE (3–12 µg mL^−1^) and DP (300–1200 µg mL^−1^), with R^2^ values exceeding 0.99. Trueness, assessed in terms of percentages of recovery, was also found to be satisfactory for both analytes, with recovery rates of 106 ± 8% for EE and 93 ± 9% for DP. Furthermore, intra-day and inter-day precision studies yielded relative standard deviation values below 6% for both analytes. In terms of sensitivity, the instrumental limits of detection were 0.25 µg mL^−1^ for EE and 6.6 µg mL^−1^ for DP, and the instrumental limits of quantification 0.82 µg mL^−1^ for EE and 22 µg mL^−1^ for DP. The method was successfully applied to the analysis of contraceptive tablets from three different pharmaceutical companies. No differences were observed between the measured and the declared amount of active principle per tablet, demonstrating the applicability of the procedure. In addition, a stability study conducted on both the standards and sample extracts demonstrated that they can be stored at room temperature for a minimum period of seven days.

## 1. Introduction

One of the most commonly used contraception methods nowadays consists of the administration of an estrogen and a progestogen, which together inhibit the ovulation process. Two synthetic versions of these hormones that are typically combined in contraceptive tablets are 17α-ethinyl estradiol (EE) and drospirenone (DP, 6β,7β:15β,16β-dimethylene-3-oxo-17α-pregn-4-ene-21,17-carbolactone).

To minimize their known adverse side effects, such as hypercoagulability, and ensure an appropriate balance between efficacy and safety, the hormone dosage per tablet is carefully optimized. Currently, DP and EE can be administered to women in two different formulations. One contains 0.02 mg of EE and 3 mg of DP (DP/20EE), while the other contains 0.03 mg of EE and 3 mg of DP (DP/30EE) per tablet. Another difference between these treatments relates to the dosage strategy: DP/30EE is administered for 21 consecutive days, followed by 7 days of rest, whereas DP/20EE is administered for 24 consecutive days, followed by 4 days of rest [1,2,3].

Due to the small doses used and the long duration of the hormonal treatments (years, even decades), accurately quantifying the amount of the active principles is crucial in the preparation of contraceptive pills [4]. Most of the analytical methods published up to date for quantifying EE and DP in tablets are based on liquid chromatography (LC) with spectrophotometric detection, either with a single UV detector [5,6], or with a UV detector for DP and a fluorescence detector (FD) in series for EE [4]. In fact, LC–UV is the preferred technique for DP quantification [7], while both LC–UV [8,9,10,11] and LC–FD [12] have been applied to contraceptive formulations combining EE with progestins other than DP.

The use of mass spectrometry (MS) has been gaining attention as a complementary detection technique, also for hormones. However, MS-based methods have primarily been applied to determine EE and DP in complex biological and environmental samples, in line with the need of an increased selectivity and sensitivity. For instance, liquid chromatography coupled to mass spectrometry (LC–MS) has been used to determine DP in plasma [13], blood [14], and water [15], whereas gas chromatography coupled to mass spectrometry (GC–MS) has been applied to separate and quantify EE and other related hormones in urine [16], cattle hair [17], water [18,19,20,21], and sediments [22]. When determined via GC–MS, hormones are usually derivatized via silylation to increase their volatility and reduce their polarity, thereby improving the physicochemical properties for their subsequent GC analysis. However, this step involves longer analysis times, the use of organic (sometimes toxic) reagents, and a higher sample manipulation that may increase the uncertainty of the obtained results. There are only a few examples where EE has been determined via GC–MS without derivatization [23,24,25], but neither in combination with DP nor in pharmaceutical formulations.

In this line, and since GC–MS has never been applied to neither the analysis of EE-containing tablets nor to the determination of DP, herein we report the first GC–MS method for the simultaneous and accurate quantification of both active principles in contraceptive tablets. Unlike other methods, our procedure does not require the use of derivatizing agents. Instead, analytes are extracted by ultrasound-assisted extraction, the extract is diluted and centrifuged, and the supernatant is directly injected into the GC–MS system with a split mode regime. To correct for potential instrumental variations, calibration is performed by the internal standard method, using cholesterol as the internal standard (IS). The aim of this work is to provide a simple, accurate, and reliable method based on GC–MS as an alternative technique to LC (which has sometimes required the use of two different detectors) for the determination of EE and DP in contraceptive formulations.

## 2. Results and Discussion

### 2.1. GC–MS Optimization

The main aim of the study was to optimize and validate a GC–MS-based method, as an alternative to those based on LC–UV and/or LC–FD, for the simultaneous determination of EE and DP in contraceptive pills. With the aim of prioritizing simplicity and sustainability, no derivatization step was performed, which is a real challenge considering the polarity and boiling points of both active principles: predicted values of log K_ow_ = 3.67 (EE) and 4.02 (DP); boiling points = 411 °C (EE) and 552 °C (DP) [26].

MS detection was performed in SIM mode. Different dwell times were evaluated, and the selected optimal values were 20 ms for EE, ES, and the IS (100 ms was the optimal value observed for DP).

Chromatographic separation was performed on an HP-5MS UI capillary column (5% phenyl-methylpolysiloxane). Isothermal conditions (325 °C) were initially explored to achieve the shortest possible chromatographic run time. In addition, different programs were tested, considering different initial oven temperatures (from 200 °C to 300 °C), applying the maximum allowed ramps by the system, and maintaining the final temperature at 325 °C (the maximum recommended by the column manufacturer). No difference was observed, but in terms of precision, the program starting at 250 °C and ramping up to 325 °C at a rate of 45 °C min^−1^ yielded the best results, with less than 6% of RSD for both analytes. Under these conditions, the retention times of EE, the IS, and DP were 2.32 min, 3.19 min, and 6.15 min, respectively.

Initial studies were conducted using an injector filled with an empty straight glass liner, with the temperature set to the maximum recommended value of 275 °C. However, this setup resulted in very low injection precision, with RSD values exceeding 40%. The replacement of this liner by another one filled with deactivated glass wool improved the % RSD values considerably (less 6%). Consequently, this packing material was selected to continue with the study. The injection volume was set at 1 μL. Different split ratios (1:2, 1:10, 1:50) were tested in order to obtain a non-saturated peak for DP and, at the same time, a satisfactory peak height for EE (in contraceptive tablets, and so in our sample extracts, DP is 100 or 150 times more concentrated than EE). The best compromise was achieved with a split ratio of 1:50, which was the ratio finally selected to continue with the study.

### 2.2. Extraction Optimization

**Figure 1:**
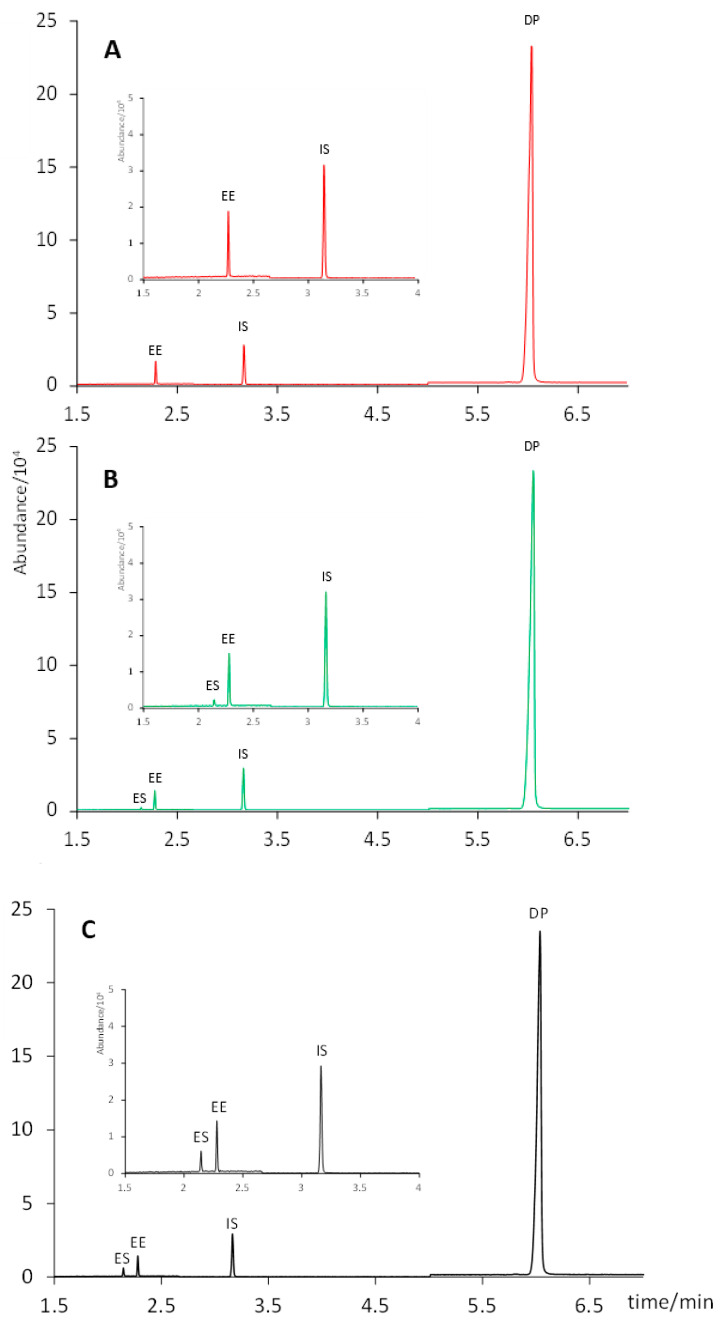
Chromatograms of the standard solutions (6 µg mL^−1^ of EE and 600 µg mL^−1^ of DP) prepared in (**A**) ethyl acetate; (**B**) methanol:ethyl acetate 1:5; (**C**) methanol.

Initially, all standard solutions used in this study were prepared in methanol due to its suitability as an organic solvent for the subsequent solid–liquid extraction of the active principles, considering their polarities. However, when these methanolic solutions were injected into the GC–MS system, we observed a partial degradation of EE to form ES (confirmed by its MS spectrum). This could be related to the reactivity of methanol at high temperatures (in the injector), which does not happen with ethyl acetate and which leads to the formation of ES from EE by oxidation of the hydroxyl moiety and liberation of the ethinyl group. In contrast, when standard solutions were prepared in ethyl acetate, no traces of ES were detected at any of the concentration levels studied during the linearity tests (Figure 1). Hence, all standard solutions were prepared in ethyl acetate hereinafter.

**Figure 2:**
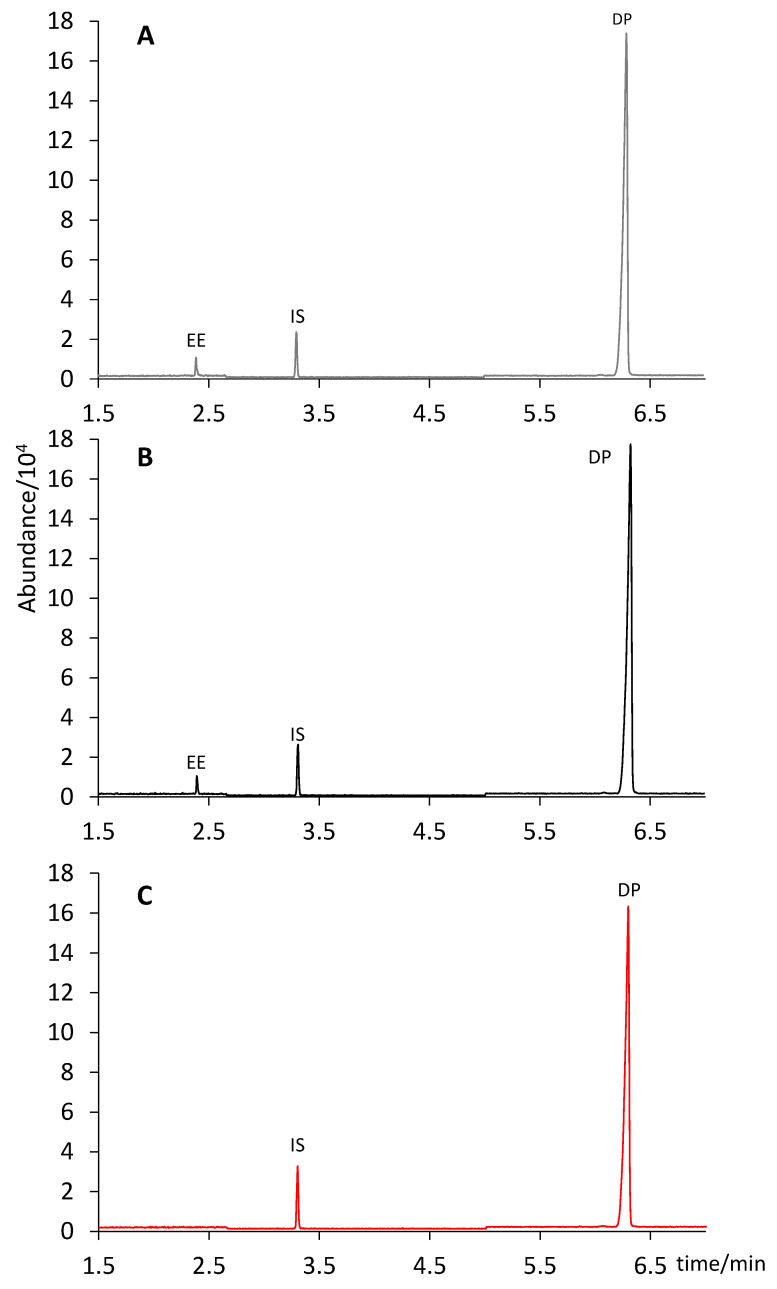
Chromatograms of the tablet extracts obtained by different extraction procedures: (**A**) extraction in 1.0 mL of methanol, evaporation to dryness and reconstitution in 5.0 mL of ethyl acetate; (**B**) extraction in 1.0 mL of methanol, made to a final volume of 5.0 mL with ethyl acetate; (**C**) extraction in 1.0 mL of ethyl acetate, made to a final volume of 5.0 mL also with ethyl acetate.

The solid–liquid extraction procedure was based on other procedures described in literature for similar formulations [4,8,27,28]. Both methanol and ethyl acetate were evaluated as extractant solvents. To this end, the mass powder equivalent to the weight of one tablet (determined by weighing 20 individual tablets, see Section 2.2) was subjected to ultrasound-assisted extraction (15 min) in 1.0 mL of either methanol or ethyl acetate. After centrifugation, the extracts in ethyl acetate (n = 3) were made up to a final volume of 5.0 mL with the same solvent. For the extracts in methanol, two approaches were considered: either diluting them to 5.0 mL with ethyl acetate (n = 3) or evaporating them to dryness and reconstituting them in 5.0 mL of ethyl acetate (n = 3). As shown in Figure 2, ethyl acetate was not able to extract EE from the solid pill. Conversely, this compound was completely recovered from the solid when using methanol as an extractant solvent. No formation of ES was observed neither when the extract was evaporated to dryness and reconstituted in 5.0 mL of ethyl acetate, nor when it was directly diluted to a final volume of 5.0 mL with ethyl acetate. Hence, the latter (and simpler) protocol was selected: ultrasound assisted extraction with 1.0 mL of methanol for 15 min, and dilution to 5.0 mL with ethyl acetate. It is worth noting that standard solutions prepared in this solvent composition (20% of methanol in ethyl acetate) still showed the formation of ES from EE (Figure 1), unlike what was observed in sample extracts. This difference could be attributed to other ingredients present in the pharmaceutical formulation, which might have prevented the oxidation from EE to ES. Thus, we kept the preparation of the standard solutions in pure ethyl acetate.

### 2.3. Method Validation

**Figure 3:**
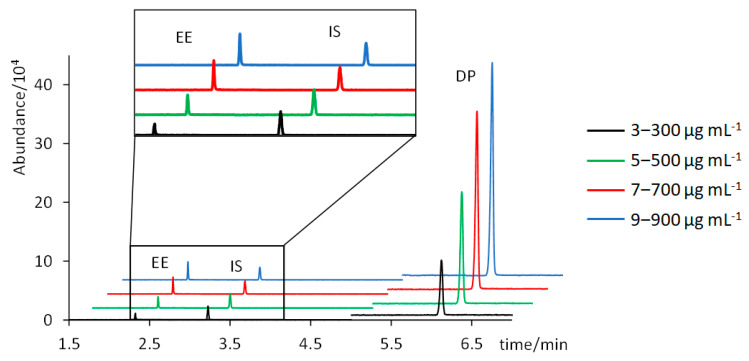
Chromatograms of standard solutions containing 3, 5, 7, and 9 µg mL^−1^ of EE and 300, 500, 700, and 900 µg mL^−1^ of DP.

Table 1 summarizes the analytical performance results of the proposed method. A good linearity was achieved for both analytes, with R^2^ values of 0.9919 for EE and 0.9902 for DP. Figure 3 shows the chromatograms of four standard solutions containing 3, 5, 7, and 9 µg mL^−1^ of EE, and 300, 500, 700, and 900 µg mL^−1^ of DP (chromatograms of intermediate concentrations have been excluded for clarity). Trueness, assessed in terms of percentages of recovery, and intra- and inter-day precision, assessed through % RSD values, were satisfactory: percentages of recovery were 106 ± 8% for EE and 93 ± 9% for DP; and % RSD values were below 4% for EE and below 6% for DP. Instrumental LODs (concentration corresponding to S/N = 3) were 0.25 µg mL^−1^ for EE and 6.6 µg mL^−1^ for DP. Instrumental LOQs (concentration corresponding to a S/N = 10) were 0.82 µg mL^−1^ for EE and 22 µg mL^−1^ for DP.

### 2.4. Analyte Stability

Analyte stability was assessed in a standard solution and in the sample extracts kept at room temperature (22 °C) for 7 days, as stated in Section 2.5.

Analyte responses at different days (analyte area/IS area, with the IS added every day just before injection) were divided by the corresponding analyte response at the beginning of the study (day 0). In the standard solution, these relative responses varied between 1.03 ± 0.03 and 1.17 ± 0.10 for EE, and between 0.95 ± 0.04 and 1.13 ± 0.16 for DP. In the tablet extracts, they ranged from 0.94 ± 0.05 to 1.00 ± 0.05 for EE, and from 0.97 ± 0.09 to 1.27 ± 0.12 for DP. These results suggest that there was no relevant degradation observed in any case, proving that both the standards and tablet extracts can be stored at room temperature for a minimum of seven days.

### 2.5. Analysis of Pharmaceutical Preparations

To test the applicability of the developed method, three contraceptive formulations from three different pharmaceutical companies were subjected to the procedure described here. Four tablets from each trademark were processed as detailed in Section 2.2 and analyzed via GC–MS as stated in Section 2.4.

**Figure 4:**
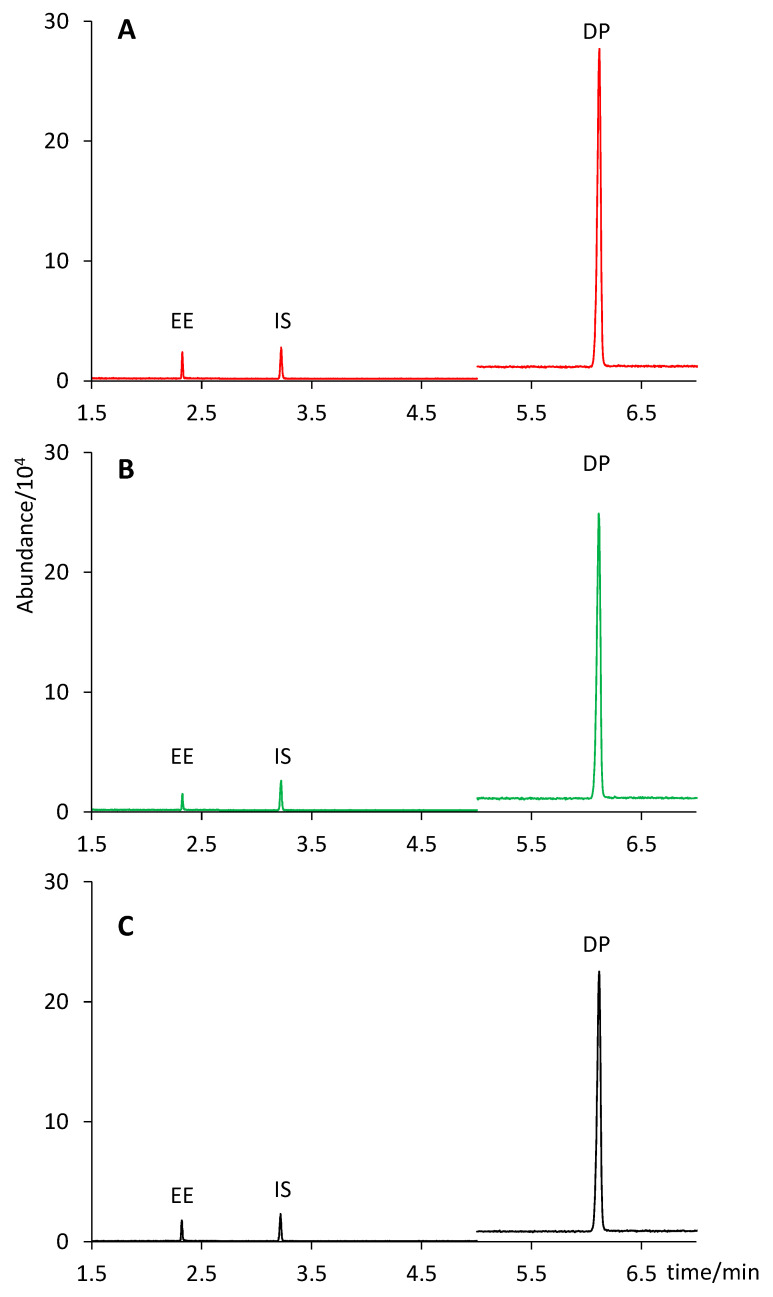
Chromatograms of (**A**) a Yasmin tablet extract; (**B**) a Stada tablet extract; (**C**) a standard solution containing 6 µg mL^−1^ of EE and 600 µg mL^−1^ of DP.

According to the pharmaceutical company, two formulations contained 0.030 mg of EE and 3.0 mg of DP per tablet (Yasmin and Drosure), whereas the third one contained 0.020 mg of EE and 3.0 mg of DP (Stada). As shown in Table 2, no significant differences were observed in any case between the content claimed by the company (considering a 10% interval according to USP [29]) and the amount predicted by our analysis with a confidence interval of 95%: Yasmin (0.027 ± 0.001 mg of EE and 2.93 ± 0.08 mg of DP); Drosure (0.028 ± 0.001 mg of DP and 2.88 ± 0.08 mg of DP); Stada (0.018 ± 0.001 mg of EE and 2.84 ± 0.08 mg of DP). Figure 4 shows a comparison of the extract chromatograms of two different tablet formulations (Yasmin and Stada) with that of a standard solution containing 6 µg mL^−1^ of EE and 600 µg mL^−1^ of DP (concentration equivalent to that of an extract from a tablet containing 0.030 mg of EE and 3.0 mg of DP).

## 3. Materials and Methods

### 3.1. Chemicals and Standard Solutions

Ethinyl estradiol (EE, 99.7% purity) and drospirenone (DP, 98.7% purity) were kindly supplied by a local pharmaceutical company. Cholesterol (used as IS, >99% purity), HPLC grade methanol, and ethyl acetate were supplied by Sigma Aldrich (Steinheim, Germany). Individual stock solutions of EE (1000 μg mL^−1^), DP (5000 μg mL^−1^), and the IS (1000 µg L^−1^) were prepared by dissolving the accurately weighed reference standard in either methanol or ethyl acetate. These solutions were stored in the dark at −22 °C and allowed to warm up to room temperature immediately before use. Working standard solutions were prepared daily by diluting the stock solutions in methanol or ethyl acetate to the required working concentrations.

### 3.2. Samples and Sample Preparation

Contraceptive tablets from three different brands (Yasmin (Bayer España, Spain), Drosure (EFFIK S.A., Madrid, Spain), and Stada (Stada S.L., Barcelona, Spain)), containing 0.03 mg of EE and 3 mg of DP per tablet (Yasmin and Drosure), or 0.02 mg of EE and 3 mg of DP (Stada), were purchased at a local pharmacy. The tablets used for method optimization and validation, containing 0.03 mg of EE and 3.00 mg of DP, were kindly supplied by a local pharmaceutical company. To determine the average tablet weight, twenty tablets were accurately and individually weighed. To prepare a relevant amount of homogenized pill powder (for method optimization), 60 pills were finely powdered and mixed.

Under the final working conditions, one tablet (for commercial tablets) or the amount of powder equivalent to the weight of one tablet (0.0828 g, for method optimization tablets) was accurately weighed into a 5.0 mL volumetric flask. IS (0.25 mg in ethyl acetate) was added to the powder and the mixture was allowed to dry and age overnight in a fume hood, under light-protected conditions. The next morning, the dried mixture was suspended in 1.0 mL of methanol and sonicated for 15 min to extract EE and DP. After cooling to room temperature, the suspension was brought up to a final volume of 5.0 mL with ethyl acetate and centrifuged at 4000 rpm for 10 min. Finally, 1.0 mL of the supernatant was transferred to a glass vial for analysis.

Spiked samples, used to calculate the recovery and relative standard deviation values at a high concentration level, were prepared by mixing an accurate mass of tablet powder with the required amounts of EE, DP, and IS solutions in ethyl acetate. The mixture was homogenized, allowed to age and dry overnight, and processed, as previously described for only-IS-spiked samples.

### 3.3. Instrumental Configuration

All the chromatographic analyses were conducted using an Agilent 7890A series gas chromatograph (Agilent Technologies, Santa Clara, CA, USA) interfaced to an Agilent 5978C inert XL MSD equipped with an electron ionization source (70 eV). Injection was performed using an MPS2 Multi-Purpose Sampler (Gerstel, Mülheim an der Ruhr, Germany). The inlet was fitted with a liner (71 mm × 2 mm i.d., Gerstel CIS-4) packed with deactivated glass wool. A volume of one microliter of the standard or sample solution was injected in a split mode (split ratio of 1:50), maintaining the injection port at 275 °C. The split flow was set at 20 mL min^−1^ and the septum purge flow at 4 mL min^−1^. Chromatographic separation was performed using an HP-5MS UI capillary column (30 m × 0.25 mm i.d.; 0.25 μm film thickness) supplied by Agilent Technologies. Helium (99.999% of purity, Air Liquid) was used as carrier gas at a constant flow rate of 2.0 mL min^−1^. The GC oven temperature was programmed as follows: initial temperature of 250 °C, increase at a rate of 45 °C min^−1^ to 325 °C, and held for 5.7 min (total run time of 7.4 min). The total analysis time per sample was 32.4 min: 15 min for ultrasound-assisted extraction, 10 min for centrifugation, and 7.4 min for the chromatographic analysis.

The MS system consisted of an EI source (70 eV), a single quadrupole analyzer, and an electron multiplier detector. The temperatures of the ionization source, mass analyzer, and transfer line were set at 230 °C, 150 °C, and 300 °C, respectively. The mass analyzer was operated in the selected ion monitoring (SIM) mode, acquiring one quantifier ion and two qualifier ions per compound (1 min of solvent delay). Table 3 shows the three SIM groups and the *m*/*z* values acquired for both active principles (EE and DP), the IS, and estrone (ES), an observed by-product of EE during method optimization. The selected dwell times were 20 ms (ES, EE, and IS) and 100 ms (DP). MSD ChemStation, Ver. E.02.00.493 software from Agilent Technologies was used for data acquisition, and NIST_98 database (NIST/EPA/NIH Mass Spectral Library, version 2.0) was used for identification.

### 3.4. Quantification and Method Validation

To correct for potential instrument variations, the analytes were quantified using the internal standard method. The calibration curves were prepared by diluting the stock solutions (Section 2.1) in ethyl acetate. They consisted of ten concentration levels: 3.0, 4.0, 5.0, 6.0, 7.0, 8.0, 9.0, 10.0, 11.0, and 12.0 μg mL^−1^ for EE, and 300, 400, 500, 600, 700, 800, 900, 1000, and 1200 μg mL^−1^ for DP. The IS was maintained at 50 µg mL^−1^. Instrumental LOD and LOQ values were calculated as the analyte concentration providing a signal-to-noise ratio (S/N) of 3 (LOD) and 10 (LOQ). These values were estimated from standards solutions in ethyl acetate containing 0.3 μg mL^−1^ of EE and 3 μg mL^−1^ of DP.

Trueness was assessed from recoveries (expressed as percentages) calculated as the difference between the concentrations measured in the extracts from spiked samples and in the extracts from samples containing only the IS, divided by the theoretical spiked level. To this end, six spiked and six only-IS-spiked samples were prepared: the amount of powder equivalent to the weight of one tablet (0.0828 g) was spiked with both the analytes (total mass added: 0.03 mg of EE and 3.00 mg of DP) and IS (0.25 mg), or only with IS (0.25 mg). These were allowed to age overnight, and were then extracted as detailed in Section 2.2.

Precision was assessed by calculating the relative standard deviation (% RSD) obtained from the analysis of spiked samples (0.03 mg of EE, 3.00 mg of DP, and 0.25 mg IS) and only-IS-spiked samples (0.25 mg). Intra-day precision was estimated from the analysis of six samples of each type within the same day, while inter-day precision was assessed from the analysis of four samples of each type performed in three different days over a two-week period.

Linearity was evaluated by calculating the determination coefficient (R^2^) value after plotting the peak response (analyte area/IS area) for EE and DP, versus their corresponding concentrations (range: 50–200% of the tablet theoretical concentration).

### 3.5. Stability Study

To assess the analyte stability, 50.0 mL of a standard solution containing 6 μg mL^−1^ of EE and 600 μg mL^−1^ of DP in ethyl acetate was prepared. With the same aim, 12 tablets extracts were prepared following the procedure described in Section 2.2. Both the standard solution and the sample extracts were stored in closed glass vials, protected from light and kept at a controlled room temperature (22 °C). Three aliquots from the standard solution and three tablet extracts were analyzed via GC–MS, as described in Section 2.3. at the beginning of the study (day 0) and after 1, 3, and 7 days of storage. Prior to analysis, the IS solution was added to each aliquot resulting in a final concentration of 5.7 µg mL^−1^, 570 µg mL^−1^, and 47.6 µg mL^−1^ of EE, DP, and IS, respectively. A fresh standard solution in ethyl acetate (also containing 5.7 μg mL^−1^ of EE, 570 μg mL^−1^ of DP, and 47.6 µg L^−1^ of IS) was prepared daily and analyzed together with every set of samples.

## 4. Conclusions

As stated in the introduction, the analysis of contraceptive formulations containing both EE and DP has been usually performed via LC with spectrophotometric detection, either with a single UV detector or with a UV detector for DP and a fluorescence detector (FD) coupled in series for EE detection. In terms of the speed of analysis, the typical times using LC were approximately 7.0 min [4,5,6], considering only the chromatographic run time. As an alternative, herein we report the first GC-based method for the simultaneous and accurate quantification of these two synthetic hormones in contraceptive tablets. A total chromatographic run time of 7.4 min was achieved, in the same time range than most LC methods reported in the literature. To the best of our knowledge, this is also the first time that GC coupled to MS has been applied for the determination of DP.

The sample preparation in this method is straightforward, involving an ultrasound-assisted solid–liquid extraction (15 min), followed by dilution, centrifugation (10 min), and a direct injection into the GC–MS system. Due to the high concentrations of the active principles in contraceptive tablets, no derivatization needed to be applied in this case. This eliminated the need for toxic derivatizing reagents and reduced sample manipulation and the uncertainty of the results obtained. The entire method was validated in terms of linearity, trueness, precision, and LODs and LOQs, proving to be consistent and reliable. In addition, the analysis of contraceptive formulations from different pharmaceutical companies (and containing different amounts of active principles per tablet) showed no difference between the measured and the claimed amounts of EE and DP, demonstrating its accuracy and applicability.

Finally, a stability assessment has proven that both the standards and sample extracts, prepared as stated here, can be stored at room temperature for a minimum of seven days, further establishing the practicality and convenience of our procedure.

Hence, this work has proven that GC–MS can be an alternative technique for the determination of EE and DP in contraceptive tablets, giving quality control laboratories an extra tool for content analysis within this type of preparations.

## Figures and Tables

**Table 1:** Analytical performance figures.

	EE	DP
Linearity range (µg mL^−1^)	3.0–12.0	300–1200
Regression equation	y = (0.121 ± 0.002)x + (0.01 ± 0.02)	y = (0.045 ± 0.001)x + (0 ± 1)
R^2^	0.9919	0.9902
Trueness (% recovery ± SD)	106 ± 8	93 ± 9
Intra-day precision (% RSD)	3.9 ^a^	5.4 ^a^
	2.5 ^b^	4.9 ^b^
Inter-day precision (% RSD)	2.7 ^a^	4.9 ^a^
	0.88 ^b^	5.9 ^b^
LOD (µg mL^−1^)	0.25	6.6
LOQ (µg mL^−1^)	0.82	22

^a^ Only-IS-spiked samples (0.25 mg). ^b^ Samples spiked with 0.03 mg of EE, 3.00 mg of DP, and 0.25 mg of IS.

**Table 2:** EE and DP contents (claimed and found) in tablets from different pharmaceutical companies.

Pharmaceutical Formulation	Active Principle Content (mg/tablet)	
	Claimed ^a^	Found ^b^
Yasmin		
EE	0.030 ± 0.003	0.027 ± 0.001
DP	3.0 ± 0.3	2.93 ± 0.08
Drosure		
EE	0.030 ± 0.003	0.028 ± 0.001
DP	3.0 ± 0.3	2.88 ± 0.08
Stada		
EE	0.020 ± 0.002	0.018 ± 0.001
DP	3.0 ± 0.3	2.84 ± 0.08

^a^ Amount stated by the company with a 10% interval, according to the USP (USP 35 NF, 2019, US PHarmacopeial Convention, Inc. Rockville, MD. USA). ^b^ Amount predicted with a confidence interval of 95%.

**Table 3:** Retention times, *m*/*z* values selected for quantification (in bold), and confirmation of the active principles (EE and DP), the IS and an EE by-product (ES).

Compound	t_R_/min	Quantifier and Qualifier/*m*/*z* Values	SIM Group
ES	2.18	**270**, 146, 185	1
EE	2.32	**213**, 296, 160	
IS	3.19	**275**, 386, 301	2
DP	6.15	**255**, 366, 117	3

## Data Availability

Not applicable.

## Sample Availability

Samples of the compounds are not available from the authors.

## Abbreviations

CIS-4	Cooled Injection System 4
DP	Drospirenone
DP/20EE	A formulation of 0.02 mg of EE and 3 mg of DP per tablet
DP/30EE	A formulation of 0.03 mg of EE and 3 mg of DP per tablet
EPA	Environmental Protection Agency
ES	Estrone
EE	17α-ethinyl estradiol
FD	Fluorescence detector
GC–MS	Gas Chromatography–Mass Spectrometry
i.d.	Inner Diameter
IS	Internal standard
LC–MS	Liquid Chromatography–Mass Spectrometry
LOD	Limit of Detection
LOQ	Limit of Quantification
MPS2	Multi-Purpose Sampler 2
MS	Mass Spectrometry
NIH	National Institutes of Health
NIST	National Institute of Standards and Technology
RSD	Relative Standard Deviation
SIM	Selected Ion Monitoring
UV	Ultraviolet
